# Interspecific Variation between the American *Quercus virginiana* and Mediterranean *Quercus* Species in Terms of Seed Nutritional Composition, Phytochemical Content, and Antioxidant Activity

**DOI:** 10.3390/molecules26082351

**Published:** 2021-04-18

**Authors:** José Valero-Galván, Raquel González-Fernández, Jesús V. Jorrin-Novo

**Affiliations:** 1 Departamento de Ciencias Químico-Biológicas, Instituto de Ciencias Biomédicas, Universidad Autónoma de Ciudad Juárez, Ciudad Juárez, Chihuahua C.P. 32310, Mexico; 2 Agroforestry and Plant Biochemistry, Proteomics, and Systems Biology, Department of Biochemistry and Molecular Biology, ETSAM, University of Cordoba, UCO-CeiA3, 14014 Cordoba, Spain; bf1jonoj@uco.es

**Keywords:** *Quercus* seeds morphometry, chemical seeds composition, clustering analysis

## Abstract

This study aimed to evaluate a complete nutritional composition in the seeds *Quercus virginiana* to compare this nutritional composition with three Mediterranean *Quercus* species. We analyzed the seed morphometry, proximate composition, phytochemicals, and antioxidant capacity. The seed of *Q. virginiana* presented the smaller seed size and weight, while *Q. suber* presented the highest values. Moreover, *Q. virginiana* seeds showed the highest amounts of sugar and total lipids, digestibility, energy, palmitic acid, and stearic acid. On the other hand, *Q. virginiana* seeds showed the lowest values of linoleic acid. Moreover, *Q. coccifera* seeds presented the highest total phenolics and flavonoids contents and antioxidant activity. The clustering analysis revealed a significant similarity in seed morphometry and nutritional composition between the Mediterranean *Q. ilex* and *Q. suber*, grouping with the American *Q. virginiana*, but to a considerable distance; by contrast, the Mediterranean *Q. coccifera* was the most distant in the clustering analysis. The content of phenolics and flavonoids and digestibility value were the variables that contributed to the separation to a greater extent in the clustering of the four species. The nutritional and biological activity assessment of plant seed may be considered as an essential mission to find new sustainable sources and novel chemical agents. In this sense, *Quercus* seeds may be an alternative and a competitive food source for the agri-food industry.

## 1. Introduction

Forest trees have enormous economic and ecological importance worldwide, but they will be seriously affected by climate change [1]. Principally, those species producing recalcitrant seeds will be particularly susceptible to the increasing intensity and frequency of drought episodes predicted by global climate changes [2]. In this context, one group of forest plants that could be affected is the *Quercus* genus, whose seeds are recalcitrant, and it grows in areas where models have predicted climate changes. Therefore, a better understanding of the ecophysiology, seed germination, and seed nutritional composition of these species might uphold restorative practices in the areas where *Quercus* distribute.

Oak seeds are of vital importance because these fruits have been widely used as feeding systems of many forestall regions, especially in the wild and livestock Mediterranean animals [3,4]. After all, acorn as feeds provides fatty acids and tocopherol, increasing the nutritional quality of animal meat [3,5] and expanding the impact at the social and economic level on farmers [6]. Furthermore, the *Quercus* spp. seeds could be an essential reservoir of nutrients for communities [7,8] because acorn consumption might contribute health benefits [9]. Instead, as a feed source, the oak seeds are mainly used in the southwestern continental of Europe, and acorns principally come from mixed stands or pure stands from *Q. suber* L., *Q. ilex* L. subsp. *ballota* (Desf.) Samp. *Q. coccifera* L., *Q. pyrenaica* Willd., and *Q. faginea* Lam [7,10]. However, several studies have shown differences in the seed morphological parameters (size and mass) [11,12]; the proximate and fatty acid profiles analyses [12,13,14,15,16], phytochemistry, and antioxidant activity [4,7,10,12,17]. These large variabilities depend on species and are frequently regulate by climatic conditions and soil composition. 

On the other hand, the forest area of North America could offer an extensive opportunity to find new functional nutrient sources such as acorns of oaks. In this regard, *Q. virginiana* Mitl. (live oak) is a long-lived species, with broadleaf and evergreen species, spreading from Florida to Texas and the east and north to Virginia. In Mexico, this species propagates in the northeast of the country included the Chihuahua, Coahuila, Nuevo León, and Tamaulipas states. In the last decades, this *Q. virginiana* has been transplanted into the urban ecosystem and this species has well established in cities in the North of Mexico because they are trees with the ability to withstand the wind and support the salinity of soils. Furthermore, live oak acorns are also a feeding system for wild and livestock animals both in the southeastern of the United States of America (USA) and the northeast of Mexico. However, there remain knowledge gaps regarding to a wide acorn chemical composition of live oak.

Limited studies have been carried out in *Q. virginiana*. Leaves showed a low level of digestible energy [18] and a variation of 13–17% of protein content [19]. Likewise, the acorn of *Q. virginiana* from the USA areas presented a good reserve of crude protein (4.6%), crude fat (5.8%), and crude fiber (18.6%) [20]. However, the complete characterizations of seeds are still unexplored in *Q. virginiana*. Hence, this research aimed to evaluate the seed morphometry, chemical composition, fatty acid contents, phytochemicals, and antioxidant activity from *Q. virginiana* acorns. Finally, we compared this information with previous studies described on the three most important oaks from the Mediterranean area.

## 2. Results

### 2.1. Morphometric and Chemical Composition Analysis of Seeds

Concerning the morphometric analysis, *Q. virginiana* seeds showed the smallest measurements of weight (2.1 ± 0.2), length (22.80 ± 0.09), diameter (12.90 ± 0.07), coat weight (0.60 ± 0.07), and megagametophyte weight (1.57 ± 0.19). However, *Q. suber* seeds exhibited the highest values in weight (6.5 ± 1.4), length (3.5 ± 0.3), diameter (1.6 ± 0.1), coat weight (1.3 ± 0.3), and megagametophyte weight (4.3 ± 0.8) (Table 1).

On the other hand, the chemical composition of ash, protein, lipids, carbohydrates, sugar, fiber, starch, digestibility, and energy also varied among the seeds of the four *Quercus* species. However, the water and fiber contents did not show variances among them (Table 2).

The acorns analysis showed that *Q. virginiana* had the highest sugar content, digestibility, and energy, while the *Q. coccifera* presented the lowest ones. Furthermore, *Q. ilex* and *Q. virginiana* exhibited the highest total lipids content, while *Q. suber* showed the lowest ones. Moreover, starch content was the highest in *Q. coccifera* and the smallest in *Q. virginiana*. *Q. suber* had the highest percentage of protein and ash, while the *Q. coccifera* presented the lowest ones. *Q. coccifera* showed the highest quantity of carbohydrates, whereas the *Q. virginiana* and *Q. ilex* presented the lowest ones.

### 2.2. Fatty Acid Determination of Seeds

The seed analysis of fatty acid composition by NIRS showed a significant variation in the stearic, linoleic, and palmitic acid contents (Table 2). *Q. coccifera* and *Q. virginiana* exhibited the highest palmitic acid contents, while *Q. suber* presented the lowest ones. Besides, seeds of *Q. virginiana* presented the higher values in stearic acid and *Q. coccifera* the lowest ones. On the other hand, seeds from *Q. virginiana* showed the lowest amounts in linoleic acid, and *Q. coccifera* presented the highest ones.

### 2.3. Phytochemical Analysis and Antioxidant Activity of Seeds

Significant differences were observed for total polyphenolics, phenolics, and flavonoids contents in the acorns (Table 2). Total polyphenolics determined by NIRS presented a high variation in the examined seeds, ranging from 0.73% for *Q. ilex* to 2.98% for *Q. coccifera*. *Q. virginiana* showed a significant difference concerning the Mediterranean *Quercus* having an intermediate value between the lowest for *Q. suber* and *Q. ilex* and the highest for *Q. coccifera*. The total phenolics contents also varied from 25.9 to 130.5 mg GAE·g^−1^, for *Q. ilex* and *Q. coccifera*, respectively. In this case, the values for *Q. Virginiana* were similar to those determined for *Q. ilex* and *Q. suber*, being in the lowest range. The total flavonoids contents fluctuated from 9.2 to 56.3 mg CE·g^−1^, showing *Q. Virginiana*, *Q. ilex*, and *Q. suber* the littlest contents. Summarizing, *Q. virginiana* acorns presented contents in total polyphenolics, phenolics, and flavonoids more similar to those from *Q. ilex* and *Q. suber*, being in the lowest range, than those determined in *Q. coccifera* seeds, that had the highest values.

The antioxidant activity determined by 2,2-azinobis-(3-ethylbenzothiazoline-6-sulfonic acid radical scavenging (ABTS) assay showed a wide variation among seed extracts varying in a range from 157 to 1990 mmol TE·g^−1^ (Table 2). Furthermore, the ferric reducing antioxidant power (FRAP) assay measures also showed a wide variation between the seed extracts ranging from 157 to 1343 mmol TE·g^−1^. Both antioxidant capacity assays showed that *Q. virginiana* acorns exhibited an antioxidant activity similar to those from *Q. suber*, with intermediate values between the lowest presented by *Q. ilex* and the highest determined in *Q. coccifera* seeds (Table 2).

**Figure 1:**
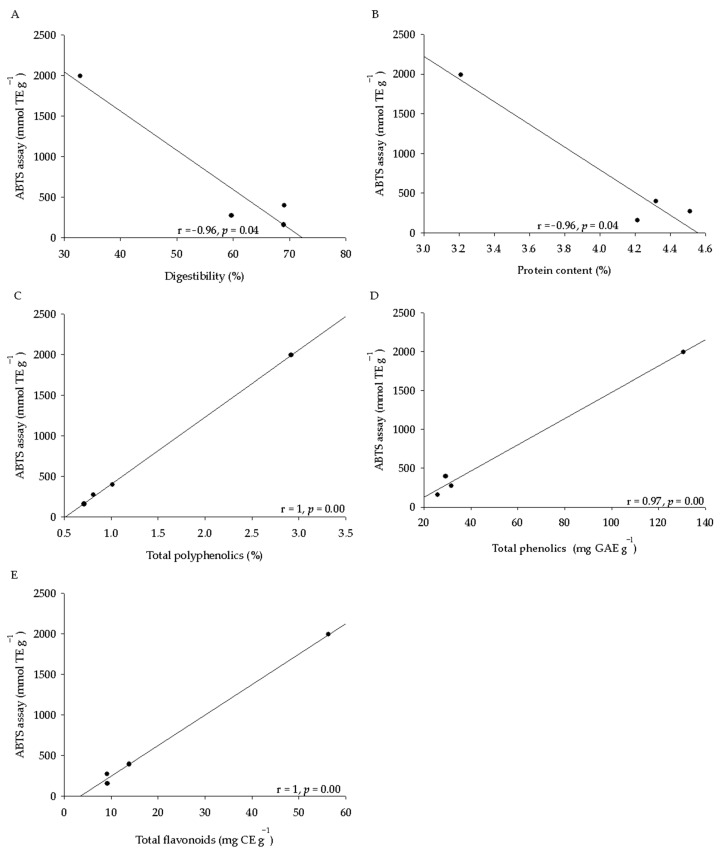
Correlation between the antioxidant activity data determined by ABTS assay and the nutritional and phytochemical contents. Correlation between antioxidant activity and digestibility (**A**), protein contents (**B**), total polyphenolics (**C**), total phenolics (**D**), and total flavonoids (**E**). Pearson’s correlation coefficient is indicated with a level of significance (*p* ≤ 0.05).

**Figure 2:**
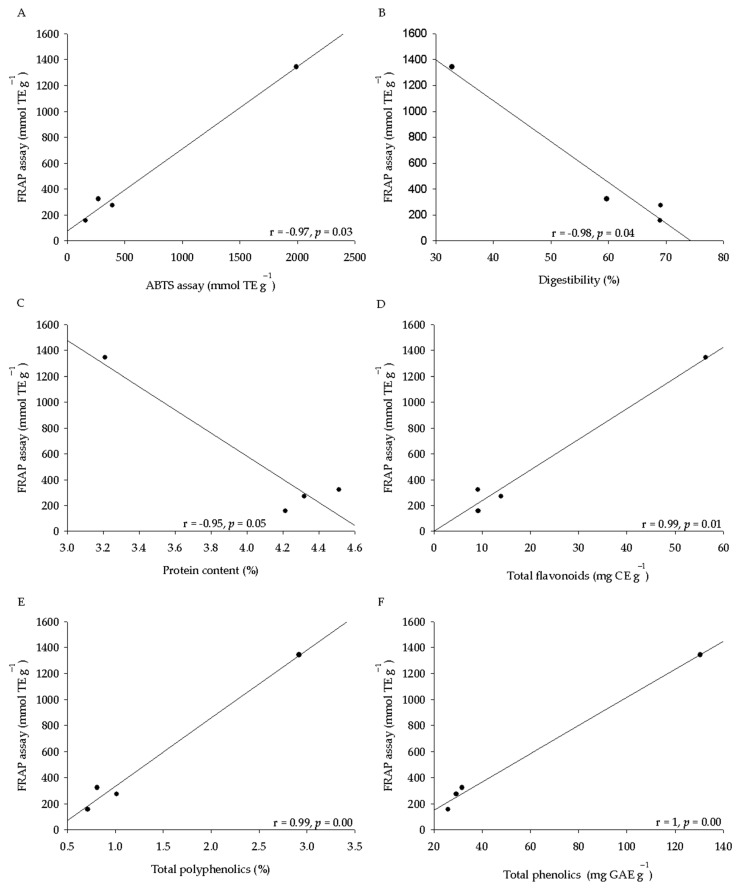
Correlation between the antioxidant activity determined by FRAP assay and the nutritional and phytochemical contents. (**A**) Correlation between the antioxidant activity determined by FRAP and ABTS assay. Correlation between the antioxidant activity determined by FRAP assay and digestibility (**B**), protein contents (**C**), total flavonoids (**D**), total polyphenolics (**E**), and total phenolics (**F**). Pearson’s correlation coefficient is indicated with a level of significance (*p* ≤ 0.05).

In the present study, we observed variation between the antioxidant activity and the species of origin of the acorn extracts and the methodology used to determine this capacity. These variations could be related to the nutritional composition and phytochemical contents. In this context, we carried out a Person’s correlation. The antioxidant activity determined by ABTS assay showed a negative correlation with digestibility (r = −0.96, *p* = 0.04; Figure 1A) and protein contents (r = −0.96, *p* = 0.04; Figure 1B) and a positive correlation with the total polyphenolics (r = 1, *p* = 0.00, Figure 1C), total phenolics (r = 0.97, *p* = 0.00, Figure 1D), and total flavonoids (r = 1, *p* = 0.00; Figure 1E). Moreover, both antioxidant activity assays showed a positively correlation with each other (r = 0.97, *p* = 0.03; Figure 2A). On the other hand, the antioxidant activity determined by FRAP assay also showed a negative association with the digestibility (r = −0.98, *p* = 0.02; Figure 2B), protein contents (r = −0.95, *p* = 0.05; Figure 2C) and a positive correlation with the total flavonoids (r = 0.99, *p* = 0.01; Figure 2D), total polyphenolics (r = 0.99, *p* = 0.00; Figure 2E), and total phenolics (r = 1, *p* = 0.00; Figure 2F).

### 2.4. Clustering Analysis

**Figure 3:**
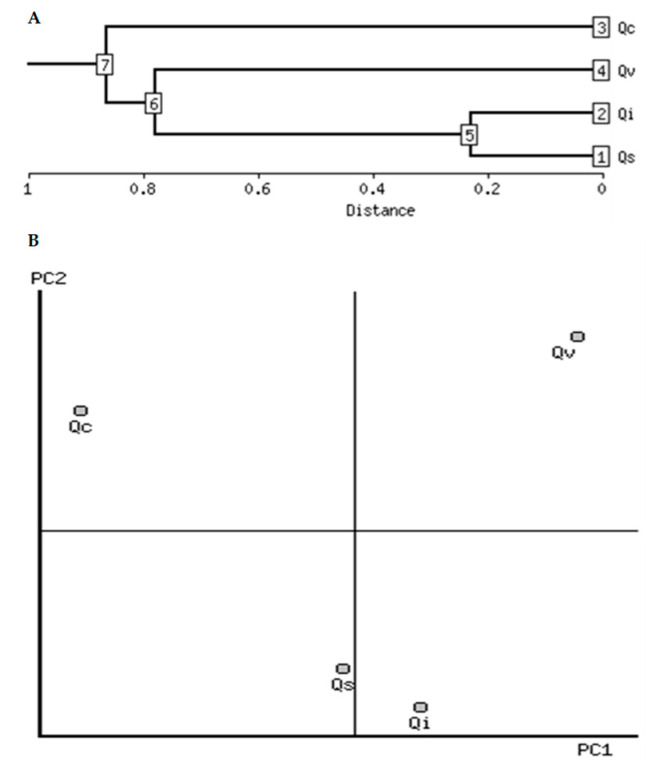
Associations between morphometric parameters and nutritional composition of the four *Quercus* species by hierarchical clustering (**A**) and PCA analysis (**B**). Qc: *Q. coccifera*, Qs: *Q. suber*, Qi; *Q. ilex*, and Qv: *Q. virginiana*.

The clustering analysis showed that the Mediterranean *Q. ilex* and *Q. suber* were the most similar for seed morphometry characteristics and the nutritional composition, whereas American *Q. virginiana* classified inside this cluster but with a considerable distance. However, the Mediterranean *Q. coccifera* was the most distant in the clustering analysis (Figure 3A). The PCA analysis determined that 98.44% of the total variability was explained by the first two components, with 51.6% and 46.8%, respectively (Figure 3B). This analysis also revealed that the American *Q. virginiana* separated from the other three Mediterranean species, being *Q. ilex* and *Q. suber* the closest species and *Q. coccifera* the farthest one (Figure 3B), consistent with clustering analysis (Figure 3A). Moreover, digestibility and total lipid contents were correlated positively to the PC1. These two variables showed a tendency to accumulate in *Q. ilex* and *Q. virginiana* seeds. Additionally, the antioxidant activity (by FRAP assay), and the phenolics and flavonoids contents were correlated negatively to PC1 (Table 3). These results confirm that *Q. coccifera* showed higher values in these three characteristics (Table 3).

## 3. Discussion

Certain studies have suggested that desirable seed characteristics are a good indicator of the forest tree success from *Quercus* spp. in reforestation plans; for example, seed size has been correlated with the increased seedling growth and root and shoot size, improving the seedling performance [21,22]. We observed a variation in the seed morphometry characteristics among the four *Quercus* species (Table 1). The morphometry analysis of acorns from *Q. coccifera, Q. suber*, and *Q. ilex* subsp. ballota collected from the Cáceres province, Extremadura (in the east of Spain) showed different data from our study [17], for which we collected the acorns from Mediterranean *Quercus* in the Córdoba province (in the south of Spain). These authors found that *Q. ilex* had a higher weight (8.7 g), length (4.14 cm), and width (1.87 cm) compared with those from *Q. suber* (7.69 g, 3.65 cm, 1.77 cm, respectively) and *Q. coccifera* (4.1 g, 3.1 cm, and 1.5 cm, respectively) [17]. In a similar study, seed morphometry from *Q. suber* and *Q. ilex* from the same eastern Spanish region also showed values different from those found in our study [10]. In this case, *Q. ilex* presented a higher seed and megagametophyte weight (11.9 g and 9.5 g, respectively) but a lower coat weight (1.8 g) compared with *Q. suber* (9.8 g, 6.8 g, and 2.1 g, respectively). Some elements such as maturity stage, seed location in the tree and harvest time, environmental conditions from the forest areas, and the tree species are relevant characteristics that may affect the morphometric features and chemical composition modulation of *Quercus* spp. [12].

Some studies have shown an increasing interest in the chemical characterization of wild food plants to generate new alternatives to agricultural fruits or as a new ingredient for the food industry [8]. Oak seeds have been acknowledged for their high importance as animal feeding components, but now their chemical composition has extended the interest to be incorporated in human nutrition [7,8]. Our results showed a statistically significant variation in the nutritional composition of the seed of the different *Quercus* species examined (Table 2). The water content determination showed no significant changes among the acorns from the four *Quercus* species that oscillated between 71.6% (*Q. suber*) to 74.3% (*Q. ilex*). These values were higher than those found in seeds collected in a forest located in the Trás-os-Montes region (Portugal), where the acorns from *Q. ilex* and *Q. suber* presented 30% and 46%, respectively, in water content [8]. In our study, ash content also varied from 1.6% (*Q. virginiana*) to 1.8% (*Q. suber*). These results of *Q. virginiana* seeds were lower than those determined in seed collected from the USA areas, where ash percentage was 1.8% [20]. Similar results for the ash content were observed in *Q. suber* seeds collected in a forest area located in Portugal, in which the ash content was 2.5% [8]. However, the ash content for *Q. ilex* seeds collected in the south of Spain was higher (1.7%) than in *Q. ilex* seeds collected in Portugal (1.2%) [8].

*Q. virginiana* seeds presented similar protein content (4.3%) that in seeds sampled from the USA areas (4.6%) [20]. Pasqualone et al. [7] found that the protein content of seeds collected in a forest located in eastern Algeria was lower in *Q. ilex* (3.1%) and *Q. suber* (3.3%) than values measured in our study (Table 2). However, protein content determined for *Q. coccifera* (4.5%) was mildly higher than those found in our research. The total lipids determined in the seeds of *Q. virginiana* were higher than in those sampled from the USA areas, in which total fat presented the content of 5.8% [20]. Pasqualone et al. [7] also showed similar values of total lipids of seed from *Q. ilex* (7.8%), *Q. suber* (8.6%), and *Q. coccifera* (8.9%) than those obtained in our study from *Q. suber* (8%), but these were lower than those determined for *Q. ilex* (12.9%) but higher than those found in *Q. coccifera* (2.9%) (Table 2).

Carbohydrates contents obtained in this study varied from 81% (*Q. ilex*) to 92% (*Q. coccifera*). These results were similar to those found for *Q. faginea* (89%), *Q. ilex* (87%), *Q. nigra* (92%), and *Q. suber* (86%) from Portugal [23]. Pasqualone et al. [7] also found a similar carbohydrate contents in *Q. ilex* (78%), *Q. suber* (80%), and *Q. coccifera* (80%) seeds collected in eastern Algeria being mildly lower than the values determined in our study (Table 2). We found no significant differences in acorns from the four species regarding the fiber contents (Table 2). However, a variation in the fiber content was observed in different morphotypes of *Q. ilex* acorns (2.08–2.28%) from the south of Spain [12]. Furthermore, fiber content found in the seed from *Q. virginiana* was lower (Table 2) than those determined in samples from the USA areas, in which the crude fiber was 18.6% [20]. Pasqualone et al. [7] also showed that the fiber content found in seeds from *Q. ilex* (11.2%), *Q. suber* (7.7%), and *Q. coccifera* (7.1%) were mildly highest than values determined in our study (Table 2). The sugar contents varied from 6% (*Q. ilex*) to 11% (*Q. virginiana*), finding similar results in different morphotypes of *Q. ilex* acorn (5–11%) from the south of Spain [12]. Likewise, we observed a variation from 57% (*Q. virginiana*) to 63% (*Q. coccifera*) in the starch contents, with these being similar results to those determined in different acorn morphotypes from *Q. ilex* (53–61%) from the south of Spain [12].

In this study, digestibility varied from 32% (*Q. coccifera*) to 69% (*Q. virginiana* and *Q. ilex*). However, in different acorns morphotypes from *Q. ilex* from the south of Spain, the digestibility was minor (57–65%) [12] than our results from *Q. virginiana* and *Q. ilex*. Energy content in our study varied from 444 kcal/100 g (*Q. coccifera*) to 490 kcal/100 g (*Q. ilex*), being higher than those reported for *Q. faginea* (318 kcal/100 g), *Q. ilex* (412 kcal/100 g), *Q. nigra* (381 kcal/100 g), and *Q. suber* (382 kcal/100 g) from Portugal [23] and similar than those obtained in different acorn morphotypes from *Q. ilex* (469–510 kcal/100 g) from the south of Spain [12].

The fatty acid composition of oak seeds has shown a high variation in the percentages of saturated, monounsaturated, and polyunsaturated fatty acids. In this study, a high content of oleic acid (>66% of total fatty acids) was observed, followed by linoleic acid (>13%), palmitic acid (>12%), and stearic acid (>2%). These results agree with those determined in seeds of three oak species located in the Extremadura forest area in the east of Spain in which reflected a high content of oleic acid (>63%), linoleic acids (>14%), and palmitic acid (>10%) [10]. Furthermore, our results did not show statistically significant differences among the four oak species analyzed in the oleic acid content (Table 2). Cantos et al. [10] found similar contents of this fatty acid in acorns from *Q. ilex* (67%) and *Q. suber* (63%). However, Akcan et al. [17] detected similar content in oleic acid in *Q. suber* (56.3%) and *Q. coccifera* (48%) but significant differences comparing to *Q. ilex* (65.8%) in seeds from Cáceres region in the east of Spain. We found a variation in the linoleic acid analysis from 13.4% (*Q. virginiana*) to 19.8% (*Q. coccifera*). The linoleic acid content in acorns from *Q. suber* (20.7 %) and *Q. coccifera* (25.4 %) was mildly higher than those found in our study, but the values from *Q. ilex* (14.17%) were similar to our results [17]. The palmitic acid analysis showed a variation from 12.5% (*Q. suber*) to 16.9% (*Q. coccifera*). Our determinations were similar to those found for acorns from *Q. suber* (14.3 %), *Q. ilex* (12.8%), and *Q. coccifera* (16.2%) from Cáceres region in the east of Spain [17]. Finally, the stearic acid content ranged from 2.8% (*Q. coccifera*) to 4.3% (*Q. virginiana*), being similar to the content of this fatty acid found in *Q. suber* (2.7%), *Q. coccifera* (3.3%), and *Q. ilex* (4.03%) from Cáceres region in the east of Spain [17].

Phytochemicals are an essential group of bioactive molecules found in plant seeds and exhibit various biological activities when these are included in the diet [8]. Although previous data for these species scarce of, our results were higher than the total phenolics detected in seeds from *Q. ilex* (691 mg GAE·100 g^−1^), *Q. suber* (785.9 mg GAE·100 g^−1^), and *Q. coccifera* (1017.4 mg GAE·100 g^−1^) from an eastern Algeria forest [7] (Table 2). In the same way, the total flavonoid contents also were higher than those determined in acorns from *Q. ilex* (102.5 mg GAE·100 g^−1^), *Q. suber* (63.3 mg GAE·100 g^−1^), and *Q. coccifera* (119.4 mg GAE·100 g^−1^) [7] (Table 2). Furthermore, we determined the total polyphenolics by NIRS and total phenolics by Folin-Ciocalteu assay. To verify that the data obtained by both techniques were comparable, we performed a Pearson’s correlation analysis, which showed a high positive correlation between both methodologies used and the origin species (r = 0.99; *p* < 0.00). Thus, according to both methods for determining phenolics contents, acorns from *Q. coccifera* had the highest values, while those from *Q. ilex* had the lowest ones.

Total antioxidant activity was carried out using both FRAP and ABTS assays. Our results showed a variation in the averages associated with the oak species and the method used to determine these activities. Seeds from *Q. coccifera* presented the highest antioxidant activity determined by both assays (Table 2). These results agreed with those found in acorns from *Q. ilex* (17.20 μmol TE·g^−1^), *Q. suber* (32 μmol TE·g^−1^), and *Q. coccifera* (35.2 μmol TE·g^−1^) from an eastern Algeria forest [7]. Some studies have established that phenolics and flavonoids are present in an extract may be related to a high antioxidant capacity because these compounds are involved in the scavenging activity against oxidant radicals [8]. Our results showed that both ABTS and FRAP assay had a direct correlation with the total polyphenolics, total phenolics, and total flavonoid contents (Figure 1C,D and Figure 2D–F). However, these results were in contrast to those found in some *Quercus* spp., where the antioxidant activity of the different acorn extracts evaluated with the 2,2-diphenyl-1-picrylhydrazyl radical (DPPH) and ABTS assays did not demonstrate a direct correlation between the total polyphenolics and the antioxidant capacity [10].

## 4. Materials and Methods

### 4.1. Plant Material

*Q. virginiana* seeds were collected in the Autonomous University of Ciudad Juarez, Ciudad Juárez, Chihuahua, México (Latitude: 31°44′46.5″ N and Longitude: 106°26′36.9″ W), where *Q. virginiana* is used as ornamental plant due to the ability to withstand the wind and salinity tolerance. In this region, the climate is arid, with an average annual temperature of 18.9 °C, and an average annual rainfall of 331.8 mm (based on 59-year data from the Mexican National Meteorology Service (https://smn.conagua.gob.mx/es/climatologia) (accessed on 25 March 2021)). For taxonomy identification, leaves, flowers, and acorns of *Q. virginiana* were collected and collated with the plant material deposited (RCD 7073 *Q. virginiana* Mill) in the herbarium of plant of México located in the Autonomous University of Ciudad Juarez, Ciudad Juárez, Chihuahua, México.

The acorns from the three Mediterranean *Quercus* (*Q. suber*, *Q. ilex*, and *Q. coccifera*) were collected in the Sierra Morena of Córdoba in the south of Spain (Latitude: 37°54′16.2″ N and longitude: 4°52′26.5″ W). In this region, the climate is the Mediterranean, with an average annual temperature of 18 °C, and an average annual rainfall of 600 mm [based on 40-year data from the Spanish National Institute of Meteorology (http://www.aemet.es/es/serviciosclimaticos) (accessed on 25 March 2021)]. These three species are well widespread in this area, being *Q. ilex,* the most abundant species followed by *Q. coccifera*, whereas *Q. suber* found in the wettest areas [24]. Both *Q. suber* and *Q. ilex* species have great economic importance since their acorns are the major food source for certain animal species vital to the region’s economy (pigs, bulls, and deer). Moreover, *Q. suber* is used by cork production. The vegetation of the zone is thermophilic, with a mixture of *Q. ilex*, *Q. coccifera*, and an equitably well-preserved population of *Q. suber*. For taxonomy identification, the floras and monographs of the family, genera, and species were used [24]. Further, these *Quercus* species were identified in the field using the whole-tree silvic method, which is analogous to the standard selection method used by forester [24].

All seeds were collected during December 2014 on the same day based on their presentation of maximum seed maturation color. In both places, ten trees located at 25 m spaced out apart from each other were selected, and 50 acorns per each tree allocated in all tree’s crowns were collected. Undamaged and homogeneous acorns were taken to the lab, bleach disinfected, and were stored dry into an airtight polyethylene bag, vacuum packed, and kept at −20 °C according to Bonner and Vozzo [25].

### 4.2. Acorn Morphometry Study

**Figure 4:**
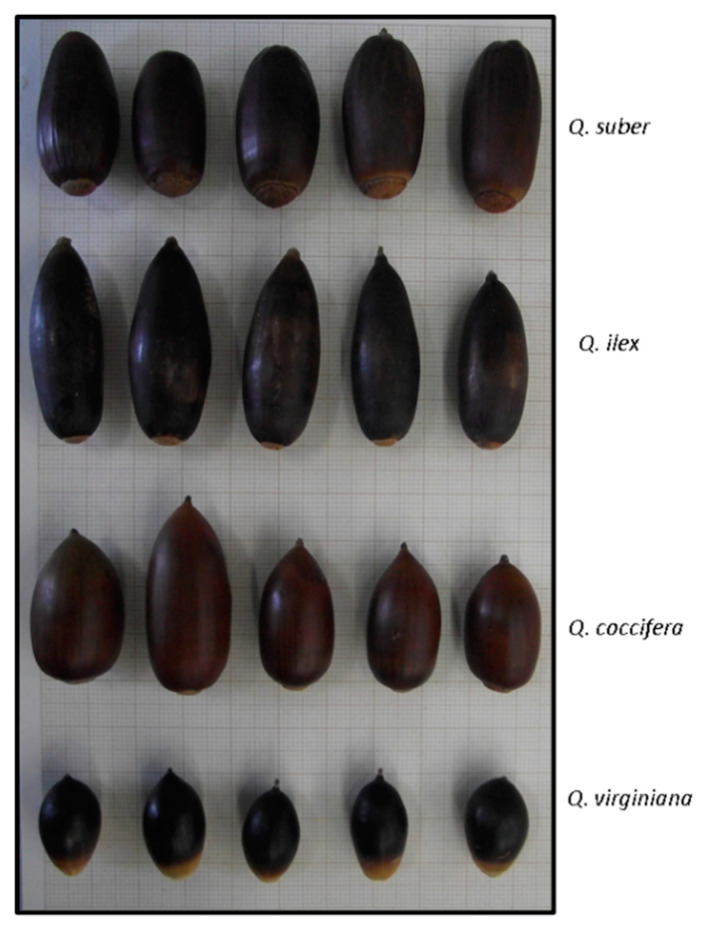
Seed variation of four *Quercus* species.

A total of 20 healthy acorns were randomly selected to measure acorn morphometry parameters from *Quercus* species (Figure 4). The weights were measured employing an analytical balance (Mettler Toledo, AJ150). For megagametophyte and coat weights, each seed was manually scarified (the seed coat of the acorns was removed using a knife by making transversal and longitudinal cuts) and weights corresponding to full seed, megagametophyte, and coat were separately measured. On the other hand, shelled and unshelled seeds were photographed, images were analyzed with the ImageJ software (ImageJ, Bethesda, MD, USA), and the diameter, length, perimeter, and area were determined.

### 4.3. Chemical Analysis

To determine the seed chemical composition, 60 seeds of each species were scarified (the seed coat of the acorns was removed using a knife by making transversal and longitudinal cuts) and the megagametophytes were pulverized using a blade mill (Moulinex, AD56 42, Lyon, France). 2 g of sample was dried at 103 °C for 24 h using a ventilation oven, later the samples were weighed, and the values were employed to determine the water content. The remaining sample was dried in a forced-air drier at 45 °C for 48 h. Next, the samples were homogenized using a Waring Blender (Waring Products, LB20E, New Hartford, CT, USA) and sieved (1 mm) to obtain a fine homogeneous flour. Five replicates from the homogeneous flour samples by each species were analyzed in the NIRS Service from the University of Córdoba (Spain) using the methodology reported by Valero et al. [13]. After NIR spectra were collected and analyzed, data were used to define the relationship to the reference characteristics data [13]. With these data, the total protein, sugars, ash, starch, fiber, fat, linoleic acid (C18:2), palmitic acid (C16:0), stearic acid (C18:0), oleic acid (C18:1), energy, digestibility, and total polyphenolics were determined by using the appropriate calibration equations that were obtained from samples of *Quercus ilex* subsp. *ballota* [13]. Total carbohydrates were calculated as the difference between 100 g and the sum of the contents obtained for ash, crude fat, and protein according to the AOAC [26].

### 4.4. Total Phenols, Total Flavonoids, and Antioxidant Activity

Seed standard extracts were obtained according to the methodology described by Kahkönen et al. [27]. In brief, 0.1 g of powders were weighed independently for each species and the sample was added to a screw-cap tube and was extracted using 5 mL of ethanol 80% in water. The tube was sonicated for 15 min, centrifuged at 4000 rpm for 15 min and the supernatant was take in into a new tube. The pellet was used again to obtain a new extract as described above and both supernatants were adjusted to 10 mL of volume.

#### 4.4.1. Total Phenolics and Total Flavonoids

Total phenolics were determined according to the methodology defined by Georgé et al. [28]. Then, 100 µL of the extract, 500 µL of Folin-Ciocalteu reagent, and 400 µL of Na_2_CO_3_ were mixed and incubated at 50 °C for 15 min. After that, the samples were cooled, placed in a microplate well, and the absorbance was taken at 740 nm on a Bio-Rad xMark Plus microplate reader equipped with Microplate Manager MPM 6.0 software (Bio-Rad, Tokyo, Japan). The total phenolic quantification was measured using a gallic acid curve as standard, and data were presented as mg gallic acid equivalents (GAE) g^−1^ of dry weight.

Total flavonoids were determined according to the methodology defined by Georgé et al. [28]. 500 µL of extract, 2 mL of water, 150 µL of NaNO_2_ (5%), 150 µL of AlCl_3_ (10%), and 2 mL of 0.5 M NaOH were mixed and stored for 30 min at room temperature. After that, the samples were placed in a microplate well, and the absorbance was collected at 510 nm. The quantification of total flavonoid contents was determined using a catechin curve as standard, and data were presented as mg catechin equivalents (CE)·g^−1^ of dry weight.

#### 4.4.2. Antioxidant Activity Quantification

The antioxidant activities by Ferric reducing antioxidant power (FRAP) and 2,2’-azinobis (3-ethylbenzothiazoline-6-sulfonate) (ABTS) assays were determined using the methodology according to Thaipong et al. [29] and modified by Moreno-Escamilla et al. [30]. In brief, in a test tube, 100 µL of the extract and 300 µL of water were mixed. Afterward, in a microplate well, 24 µL of the mixture and 180 µL of working solution (0.3 M acetate buffer, pH 3.6; 10 mM TPTZ solution in 40 mM HCl and 20 mM FeCl_3_, at a 10:1:1 ratio) were mixed, and absorbance was read at 595 nm every 30 s for 30 min.

ABTS and potassium persulfate were mixed in distilled water and allowed to react for 16 h to generate the ABTS^•+^ radical on the TEAC assay. A suitable volume of this solution was dissolved in ethanol to give an absorbance approaching 0.7 at 734 nm. Subsequently, 190 µL of the radical was mixed with 10 µL of sample and incubated for 1 min. Then, the absorbance was measured at 734 nm every 30 s for 6 min. In both cases, for FRAP y ABTS, a Trolox curve was used as standard, and results were expressed as mmol Trolox equivalents (TE)·g^−1^ dry weight.

### 4.5. Data Analysis

The seed morphometry and nutritional data were normality tested using the Kolmogorov–Smirnov test. Further, a one-way ANOVA and Duncan’s mean test were performed. Additionally, data were used to calculate a Pearson’s correlation. The statistical analysis was determined using the SPSS v.8.0 software (SPSS Inc., Chicago, IL, USA). Furthermore, the cluster analysis was determined using the web-based NIA array software [31].

## 5. Conclusions

In this study, we presented a close comparison between the American *Q. virginiana* and three Mediterranean *Quercus* species in terms of their morphometry and nutritional composition. Seeds of these species contained a high level of protein content, total lipids, and fatty acids that are particularly similar to those found in other oil tree crops, i.e., olive oil. Moreover, we observed a variation in the morphometric characteristics and nutritional composition in acorns from the different *Quercus* species. *Q. suber* presented the highest values in morphometric parameters and the highest contents of ash, protein, and oleic acid, while *Q. ilex* had the highest total lipid content. *Q. virginiana* showed the highest values in the measurements of sugar, digestibility and energy, and stearic acid. Furthermore, the phytochemicals content found in this study may provide beneficial effects to human health because of their biological activities, and it also raises the potential for new applications of oak acorns. Our results showed that *Q. coccifera* presented higher total phenolics, flavonoids, and antioxidant activities. Thus, oak acorns might be considered a functional food and used in food preparation, and therefore further studies to identify and characterize new oak species for acorns meal, nut, and oil production are strongly recommended.

## Figures and Tables

**Table 1:** Seed characteristics for the four *Quercus* species.

Seed Characteristics	*Q. virginiana*	*Q. suber*	*Q. ilex*	*Q. coccifera*	Anova (*p* ≤ 0.05)
Weight (g)	2.1 ± 0.2 ^a^	6.5 ± 1.4 ^c^	4.5 ± 1.0 ^b^	4.6 ± 1.6 ^b^	1.64 × 10^−11^
Diameter (cm)	1.3 ± 0.1 ^a^	1.6 ± 0.1 ^d^	1.4 ± 0.1 ^b^	1.5 ± 0.2 ^c^	1.54 × 10^−10^
Length (cm)	2.3 ± 0.1 ^a^	3.5 ± 0.3 ^c^	3.8 ± 0.4 ^d^	3.2 ± 0.5 ^b^	6.41 × 10^−17^
Coat weight (g)	0.6 ± 0.1 ^a^	1.3 ± 0.3 ^b^	1.0 ± 0.2 ^b^	1.3 ± 0.4 ^b^	3.05 × 10^−7^
Megagametophyte weight (g)	1.5 ± 0.2 ^a^	4.3 ± 0.8 ^c^	3.6 ± 0.7 ^b^	4.1 ± 0.8 ^bc^	5.83 × 10^−11^

Data were analyzed using a one-way ANOVA (*p* ≤ 0.05). The results are expressed as the mean ± SD (n = 20). Different letters (a–d) indicate a significant difference at *p* ≤ 0.05 (The analysis was performed in row of the characteristics evaluated).

**Table 2:** Chemical composition, fatty acids, total phenolics, total flavonoids, and antioxidant activity for the four *Quercus* species.

Chemical Composition	*Q. virginiana*	*Q. suber*	*Q. ilex*	*Q. coccifera*	Anova (*p* ≤ 0.05)
Water content (%)	73.5 ± 0.61 ^a^	71.6 ± 1.8 ^a^	74.3 ± 2.2 ^a^	72.3 ± 3.6 ^a^	0.15
Ash content (%)	1.6 ± 0.10 ^ab^	1.8 ± 0.0 ^c^	1.7 ± 0.1 ^bc^	1.6 ± 0.1 ^a^	0.01
Protein content (%)	4.3 ± 0.51 ^b^	4.5 ± 0.3 ^b^	4.2 ± 0.2 ^b^	3.2 ± 0.5 ^a^	0.01
Total lipids (%)	12.0 ± 1.62 ^c^	8.0 ± 1.6 ^b^	12.9 ± 0.7 ^c^	2.9 ± 0.4 ^a^	0.01
Carbohydrates (%)	82.1 ± 1.0 ^a^	85.7 ± 1.0 ^b^	81.2 ± 1.0 ^a^	92 ± 1.0 ^c^	0.01
Sugar content (%)	11.3 ± 0.91 ^c^	6.9 ± 0.4 ^ab^	6.0 ± 0.5 ^a^	7.5 ± 1.4 ^b^	0.01
Fiber content (%)	2.3 ± 0.03 ^a^	2.3 ± 0.0 ^a^	2.3 ± 0.0 ^a^	2.3 ± 0.0 ^a^	0.61
Starch content (%)	57.6 ± 1.57 ^a^	61.9 ± 1.3 ^b^	62.0 ± 1.5 ^b^	63.9 ± 1.7 ^b^	0.01
Digestibility (%)	69.1 ± 02.15 ^c^	59.7 ± 01.0 ^b^	69.0 ± 03.1 ^c^	32.8 ± 1.1 ^a^	0.01
Energy (kcal/100 g)	490.1 ± 8.6 ^c^	466.3 ± 8.2 ^b^	488.6 ± 4.6 ^c^	444.5 ± 3.9 ^a^	0.01
Palmitic acid (%)	16.4 ± 0.4 ^c^	12.5 ± 0.4 ^a^	15.3 ± 0.7 ^b^	16.9 ± 0.7 ^c^	0.01
Stearic acid (%)	4.3 ± 0.2 ^b^	3.1 ± 0.0 ^a^	3.1 ± 0.2 ^a^	2.8 ± 0.3 ^a^	0.01
Oleic acid (%)	66.0 ± 1.6 ^a^	67.9 ± 1.2 ^a^	66.5 ± 1.7 ^a^	66.5 ± 1.9 ^a^	0.01
Linoleic acid (%)	13.4 ± 2.6 ^a^	15.8 ± 1.1 ^b^	14.8 ± 1.2 ^ab^	19.8 ± 2.0 ^c^	0.01
Total polyphenolics (NIRS) (%)	1.03 ± 0.05 ^b^	0.80 ± 0.05 ^a^	0.73 ± 0.10 ^a^	2.98 ± 0.36 ^c^	0.01
Total phenolics (Folin Ciocalteau) *	29.4 ± 1.4 ^a^	31.8 ± 1.6 ^a^	25.9 ± 0.9 ^a^	130.5 ± 10.0 ^b^	0.01
Total flavonoids **	13.9 ± 1.9 ^a^	9.2 ± 1.0 ^a^	9.2 ± 1.1 ^a^	56.3 ± 3.4 ^b^	0.01
ABTS ***	394.1 ± 32.2 ^b^	271.9 ± 14.5 ^ab^	157.6 ± 19.7 ^a^	1990.0 ± 167.1 ^c^	0.01
FRAP ***	272.1 ± 29.0 ^b^	323.1 ± 24.4 ^b^	157.9 ± 18.1 ^a^	1343.4 ± 68.9 ^c^	0.01

* mg gallic acid equivalents (GAE)·g^−1^ of dry weight; ** mg catechin equivalents (CE)·g^−1^ of dry weight; *** mmol trolox equivalents (TE)·g^−1^ dry weight. Data were analyzed using a one-way ANOVA (*p* ≤ 0.05). The results are expressed as the mean ± SD (n = 20). Different letters (a–c) indicate a significant difference at *p* ≤ 0.05 (The analysis was performed in row of the characteristics evaluated).

**Table 3:** Seed characteristics that strongly contributed to total variability in the PCA.

Seed Characteristic	Log10 Change	Correlation	PCA Number	Direction	Qs	Qi	Qc	Qv
Total phenolics	−0.70	−0.88	1	-	1.5	1.4	2.1	1.4
Digestibility	0.34	0.93	1	+	1.7	1.8	1.5	1.8
Total flavonoids	−0.67	−0.75	1	-	0.96	0.96	1.75	1.14
FRAP assay	−0.79	−0.83	1	-	2.5	2.1	3.1	2.4
Total lipids	0.66	0.93	1	+	0.90	1.10	0.46	1.08

Qc: *Q. coccifera*, Qs: *Q. suber*, Qi; *Q. ilex*, and Qv: *Q. virginiana*.

## Data Availability

The data presented in this study are available on request from the corresponding author.

## Sample Availability

Samples of the compounds are not available from the authors.
